# The polymorphisms of extracellular matrix-remodeling genes are associated with pelvic organ prolapse

**DOI:** 10.1007/s00192-021-04917-5

**Published:** 2022-01-01

**Authors:** Lei Li, Yidi Ma, Hua Yang, Zhijing Sun, Juan Chen, Lan Zhu

**Affiliations:** National Clinical Research Center for Obstetric & Gynecologic Diseases, Department of Obstetrics and Gynecology, Peking Union Medical College Hospital, Chinese Academy of Medical Sciences & Peking Union Medical College, No. 1 Shuai Fu Yuan, Eastern District, Beijing, 100730 China

**Keywords:** Pelvic organ prolapse, Single-nucleotide polymorphism, Extracellular matrix synthesis and metabolism, ADAMTSs, MMPs, TIMPs

## Abstract

**Introduction and hypothesis:**

Extracellular matrix (ECM) synthesis and metabolism abnormalities may influence the pelvic supporting system and lead to the occurrence and development of pelvic organ prolapse (POP). Genetic polymorphisms of such related genes have been increasingly studied. This study aims to explore the association between the single-nucleotide polymorphisms (SNPs) of genes encoding ECM processing enzymes (a disintegrin and metalloproteinase with thrombospondin motifs [ADAMTSs]), ECM degrading enzymes (matrix metalloproteinases [MMPs]) and their tissue inhibitors of metalloproteinase (TIMPs), and POP.

**Methods:**

We conducted an association study including 48 women with POP at stages III and IV and 48 women without prolapse in Chinese groups. SNPs were identified using the target region sequencing technique. We performed Fisher’s exact tests to assess the association between SNPs and POP in the unadjusted model and logistic regression analysis in the adjusted model, adjusting for delivery and pregnancy.

**Results:**

There was a significant association between *TIMP2* SNP rs2277698 (odds ratio [OR], 0.37; 95% confidence interval [CI], 0.16–0.82; *P* = 0.015), *ADAMTS13* SNP rs149586801 (OR, 0.18; 95% CI, 0.05–0.69; *P* = 0.012), and *ADAMTS1* SNPs rs370850 and rs422803 (OR, 3.71; 95% CI, 1.35–10.15; *P* = 0.011 for both), rs402007, rs428785, rs434857, and rs445784 (OR, 2.18; 95% CI, 1.05–4.56; *P* = 0.038 for the four), and POP in the adjusted model.

**Conclusion:**

*TIMP2*, *ADAMTS13*, and *ADAMTS1* might be candidate genes for POP. Our results provide preliminarily new evidence for future investigation of these genes in the pathophysiology of POP.

**Supplementary Information:**

The online version contains supplementary material available at 10.1007/s00192-021-04917-5.

## Introduction

Pelvic organ prolapse (POP) is a common pelvic disorder among older women. It is characterized as the downward bulging of uterus, bladder, rectum, etc., urinary and fecal inconvenience, and sexual dysfunction, which negatively affect women’s quality of life and social activities [1]. The prevalence of symptomatic POP in China for postmenopausal women is 15% [2]. The lifetime risk of women undergoing POP surgery is 11–19%, and 30% patients still need a repeat operation [3]. Therefore, POP is becoming a social health and family burden. Understanding the pathogenesis of POP is necessary for developing novel prevention and intervention strategies for the clinic in the future.

The etiology of POP is complicated. Race/ethnicity, advancing age, obesity, higher parity, and menopause are known risk factors for POP. In addition, it is well demonstrated that POP has the characteristics of familial aggregation, as the risk of POP in women increases if their mothers or sisters have suffered from the disease, indicating a genetic contribution to POP [3]. Previous genetic analyses have revealed candidate genes associated with POP. These genes included collagen type I alpha (*COL1A1*) [4, 5], collagen type III alpha 1 (*COL3A1*) [6], laminin gamma-1 (*LAMC1*) [7], and matrix metalloproteinases (*MMPs*) [8], which are all enrolled in extracellular matrix (ECM) pathways. The latest review comprehensively summarized and identified genetic polymorphisms associated with POP, including steroid hormone receptor genes and collagen/elastic fiber synthesis genes [5]. Thus, ECM synthesis and metabolism abnormalities may affect the pelvic supporting system and have been considered the potential causes for POP.

MMPs are a family of multiple catabolic proteases involved in the degradation of collagen fibers and other components of ECM. Several previous studies have identified the existence of polymorphisms in the promoter regions of *MMP1*, *3*, and *9* genes, which could alter the expression of these genes and increase the risk of POP [8–11]. Chen et al. [12] detected three *MMP9* SNPs (rs3918242, rs17576, and rs2250889) and found rs17576 to be associated with POP. Wu et al. [13] further assessed eight *MMP9* SNPs in an association study and found two SNPs, rs3918253 and rs3918256, associated with prolapse. Wang et al. [14] demonstrated that *MMP10* SNP rs17435959 was associated with POP in a Chinese group of 91 cases and 172 controls. Besides these limited studies, other MMP members have not been studied in POP.

The bioactivity of MMPs is regulated by tissue inhibitors of metalloproteinase (TIMPs), which are also crucial factors of ECM remodeling. Allen-Brady et al. [15] carried out a genome-wide linkage study from 225 familial POP cases and found significant linkage on chromosome 17q25. In this region, the most highlighted gene for POP is *TIMP2*, which has been considered to be involved in connective tissue disorders and vascular diseases.

ADAMTSs, known as a disintegrin and metalloproteinase with thrombospondin motif, are a family of proteinases involved in procollagen processing. They can cleave the N-terminal of the peptide chains of procollagen molecules, which are the precursors of mature collagen [16]. Thus, ADAMTSs participate in the synthesis of ECM. Alarab et al. [17] found that ADAMTS2 was increased in the vaginal tissues of POP patients. However, there has been no research focusing on the relationship between the polymorphisms of TIMPs/ADAMTSs and POP.

As the fine balance between the synthesis and degradation of ECM components is essential to the integrity of the pelvic floor supportive structures, and given the previous limited findings on some of the MMP studies, and also given the fact that there has been no research on the association between the single-nucleotide polymorphisms (SNPs) of TIMPs/ADAMTSs and risks of POP, we have carried out a case-control association study in a group of Chinese women. We supposed that besides *MMP1*, *3*, *9*, and *10*, SNPs from other MMP members, TIMPs, and ADAMTSs would be associated with POP. Using a target region sequencing technique, we (1) confirmed the previous findings on MMPs and (2) investigated the susceptible loci of some of the other MMP, ADAMTS, and TIMP family genes for POP. We believe that these results might offer novel insights into the molecular mechanisms of POP development in Chinese women.

## Materials and methods

### Study samples

This was part of a case-control association study in which we recruited women from Peking Union Medical College Hospital (PUMCH) in Beijing from October 2016 to May 2017. Cases were POP patients diagnosed at Pelvic Organ Prolapse Quantification (POP-Q) stages III and IV from the Department of Gynecology and Obstetrics, and control women were diagnosed with no prolapse and have not suffered from prolapse surgery from the Physical Examination Center. All the participants signed the informed consents, and the study was approved by the Ethics Committee of PUMCH.

We excluded women with the well-known connective tissue diseases, including Marfan syndromes, Ehlers-Danlos syndromes, rheumatoid arthritis or scleroderma, and women with neurological diseases including sclerosis or stroke. As the genetic variants vary between different race/ethnic groups, all the samples were limited to Chinese ancestry. Finally, we recruited 48 cases and 48 controls subjected to the next genotyping. Sociodemographic data and physical information were collected as summarized in our previous study [7].

### Genotyping and SNP selection

Genome DNA was extracted from the peripheral venous whole-blood samples using a Puregene Blood Kit (QIAGEN, Hilden, Germany). The target region sequencing approach was used to achieve a comprehensive assessment of the specific genes; 1–2 μg DNA of each blood sample was sequenced by Agilent Liquid Capture System (Agilent SureSelect Custom Kit; Agilent Technologies, Palo Alto, CA, USA) at Novogene (Novogene Co., Ltd., Beijing, China) based on the Illumina HiSeq 4000 platform to provide a more than 200× deep of sequencing.

The valid sequencing data landed in a BAM file by Burrows-Wheeler Aligner (BWA) software based on the reference genome (UCSC hg19). Samtools and Picard (http://broadinstitute.github.io/picard) were used to sort the files and mark the duplicated reads. Samtools mpileup and bcftools were used to call variants, insertions, and deletions. The SNP quality control included SNPs with read depth > 4, mapping quality > 30, and the variant quality > 20. Variants with a minor allele frequency (MAF) > 5% remained. SNPs were assessed by PolyPhen-2, SIFT, MutationTaster, and CADD software, respectively, for the prediction of their functional effects. ANNOVAR was applied to provide the annotations of the position, type, and conservative prediction of the altered alleles and other information.

### Statistical analysis

We respectively conducted an unadjusted model and adjusted model to investigate the associations between SNPs and POP. Fisher’s exact tests were used for the unadjusted model, and logistic regression models were used for the adjusted model via PLINK. The multivariable models were adjusted for pregnancy and delivery as we did not match the two variables between case and control groups.

Odds ratio (OR) and 95% confidence interval (CI) were shown for each of the variants with OR < 1 indicating a protective effect and OR > 1 indicating a risk effect [18]. *P* < 0.05 was considered significant and < 0.1 to be suggestively significant. Hardy-Weinberg equilibrium was assessed using the χ2 test. The schematic diagrams of the gene structure that showed exons and introns as well as the type of the SNPs were graphed by the Exon-Intron Graphic Maker (http://wormweb.org/exonintron). The linkage disequilibrium (LD) graphs indicating r^2^ between SNPs were drawn by Haploview (Harvard Broad Institute, Boston, MA, USA).

## Results

We have described sample characteristics in our previous study. Briefly, we matched age (61.85 ± 10.66 vs. 62.73 ± 8.88 years, *P* = 0.663) and body mass index (BMI) (25.11 ± 3.83 vs. 24.52 ± 3.48 kg/m^2^, *P* = 0.434) between case and control groups. However, the case group had approximately ≥ 1 delivery (2.35 ± 1.34 vs. 1.58 ± 1.06, *P* = 0.005) and pregnancy (3.60 ± 1.89 vs. 2.50 ± 1.50, *P* = 0.023) in contrast to the control group. In the case group, 41 women (85.42%) were diagnosed at POP-Q stage III and 7 women (14.58%) were at stage IV as previously described [7].

Since the synthesis and degradation of the ECM is a dynamic process for the pelvic tissues, in this study, we considered several of the enzymes related to ECM processing (*ADAMTS1, 2, 3, 8, 13, 14*) and degradation (*MMP1, 2, 3, 8, 9, 10, 13*, and *TIMP1, 2, 3, 4*) in Chinese women. These genes are listed in Table 1. We have analyzed the association between SNPs and POP in both the unadjusted and adjusted model with adjustment for delivery and pregnancy. Among all these genes, we have genotyped five SNPs with *P* values < 0.1 in the unadjusted model and 16 SNPs with *P* values < 0.1 in the adjusted model that were considered to be significant or suggestively significant (Table S1). These SNPs were additionally described in detail in Table 2 and were all in Hardy-Weinberg equilibrium as they reached 10^−6^ [19].

**Table 1:** Genes genotyped in this study

Gene	Full name	cytoBand	Description
*MMP1*	Matrix metallopeptidase 1	11q22.2	Interstitial collagenase, and cleaves collagen I, II, and III
*MMP2*	Matrix metallopeptidase 2	16q12.2	Gelatinase A, and cleaves collagen I, II, III, IV and V, and elastin
*MMP3*	Matrix metallopeptidase 3	11q22.2	Stromelysin-1, and cleaves collagen III, IV, IX and X, laminin, and cartilage proteoglycans
*MMP8*	Matrix metallopeptidase 8	11q22.2	Interstitial collagenase, and cleaves collagen I, II, and III
*MMP9*	Matrix metallopeptidase 9	20q13.12	Gelatinases B, and cleaves collagen IV and V
*MMP10*	Matrix metallopeptidase 10	11q22.2	Stromelysins-2, and cleaves laminin, elastin and fibronectin, however, weakly collagens
*MMP13*	Matrix metallopeptidase 13	11q22.2	Interstitial collagenase, and cleaves collagen I, II and III, especially type II
*TIMP1*	Tissue inhibitor of metalloprotease 1	Xp11.3	Specific inhibitors of MMPs, involved in the degradation of ECM
*TIMP2*	Tissue inhibitor of metalloprotease 2	17q25.3	
*TIMP3*	Tissue inhibitor of metalloprotease 3	22q12.3	
*TIMP4*	Tissue inhibitor of metalloprotease 4	3p25.2	
*ADAMTS1*	ADAM metallopeptidase with thrombospondin type 1 motif 1	21q21.3	Aggrecanase, can degrade aggrecan, a cartilage proteoglycan, and can activate metalloproteases
*ADAMTS2*	ADAM metallopeptidase with thrombospondin type 1 motif 2	5q35.3	Cleaves the N-terminal propeptides from the fibrillar procollagens I and II, as well as excises lysyl oxidase
*ADAMTS3*	ADAM metallopeptidase with thrombospondin Type 1 motif 3	4q13.3	Cleaves the N-terminal propeptides from the fibrillar procollagen II
*ADAMTS8*	ADAM metallopeptidase with thrombospondin Type 1 motif 8	11q24.3	Inhibits angiogenesis
*ADAMTS13*	ADAM metalloproteinase with thrombospondin Type 1 motif 13	9q34.2	Cleaves von Willebrand Factor
*ADAMTS14*	ADAM metallopeptidase with thrombospondin Type 1 motif 14	10q22.1	Cleaves the N-terminal propeptides from the procollagen I

*MMP* matrix metalloproteinase, *TIMP* tissue inhibitors of metalloproteinase, *ADAMTS* a disintegrin and metalloproteinase with thrombospondin motif, *ECM* extracellular matrix

**Table 2:** Analysis for both unadjusted and adjusted models for some of the MMP, TIMP, and ADAMTS family genes

SNP	Allele frequency	Unadjusted		Adjusted^a^		HWE	Function	Exonic function	AA change
		*P* value	OR (95% CI)	*P* value	OR (95% CI)				
*MMP9*									
rs3918254	T (0.20)	0.163	1.90 (0.80–4.72)	0.079	2.21 (0.91–5.36)	0.454	Intronic	–	–
*MMP13*									
rs3758853	G (0.11)	0.104	2.96 (0.84–13.24)	0.070	3.02 (0.91–9.99)	0.447	Intronic	–	–
rs78356340	A (0.11)	0.104	2.96 (0.84–13.24)	0.070	3.02 (0.91–9.99)	0.447	Intronic	–	–
*TIMP2*									
rs2277698	T (0.18)	0.062	0.50 (0.25–0.98)	**0.015**	0.37 (0.16–0.82)	0.781	Exonic	Synonymous	S101S
*TIMP3*									
rs9862	C (0.71)	0.130	1.66 (0.91–3.03)	0.093	1.69 (0.92–3.13)	0.367	Exonic	Synonymous	H83H
*TIMP4*									
rs10433537	T (0.09)	0.058	4.83 (1.02–23.13)	0.078	4.16 (0.85–20.31)	0.262	Intronic	–	–
*ADAMTS1*									
rs370850	T (0.41)	0.076	1.87 (0.94–3.77)	**0.011**	3.71 (1.35–10.15)	0.00019	Intronic	–	–
rs422803	A (0.41)	0.076	1.87 (0.94–3.77)	**0.011**	3.71 (1.35–10.15)	0.00019	Intronic	–	–
rs402007	G (0.47)	0.106	1.68 (0.90–3.14)	**0.038**	2.18 (1.05–4.56)	0.140	UTR5	–	–
rs428785	G (0.47)	0.106	1.68 (0.90–3.14)	**0.038**	2.18 (1.05–4.56)	0.140	Exonic	Missense	A227P
rs434857	G (0.47)	0.106	1.68 (0.90–3.14)	**0.038**	2.18 (1.05–4.56)	0.140	Exonic	Synonymous	P32P
rs445784	T (0.47)	0.106	1.68 (0.90–3.14)	**0.038**	2.18 (1.05–4.56)	0.140	Exonic	Synonymous	V194V
rs436525	A (0.43)	0.180	1.56 (0.83–2.94)	0.090	1.86 (0.91–3.82)	0.190	Exonic	Synonymous	P500P
*ADAMTS13*									
rs149586801	T (0.07)	**0.011**	0.26 (0.07–0.78)	**0.012**	0.18 (0.05–0.69)	0.679	Intronic	–	–
rs1055432	A (0.17)	0.282	0.64 (0.29–1.37)	0.080	0.48 (0.22–1.09)	0.755	Exonic	Synonymous	T1407T
*ADAMTS14*									
rs4747097	T (0.38)	0.165	1.61 (0.88–2.98)	0.078	1.83 (0.93–3.56)	0.644	Intronic	–	–

Significant data (*P* < 0.05) are indicated in bold

*MMP* matrix metalloproteinase, *TIMP* tissue inhibitors of metalloproteinase, *ADAMTS*, a disintegrin and metalloproteinase with thrombospondin motif, *SNP* single-nucleotide polymorphism, *CI* confidence interval, *OR* odds ratio, *HWE* Hardy-Weinberg equilibrium, *AA* amino acid, *T* threonine, *A* alanine, *P* proline, *V* valine, *S* serine, *H* histidine

^a^Adjusted by pregnancy and parity

In the analysis of the MMP family genes, we have identified a trend toward significance for *MMP9* SNP rs3918254 and POP (OR, 2.21; 95% CI, 0.91–5.36; *P* = 0.079) after the adjustment, which was an intronic SNP. For other MMP members, we have identified two new *MMP13* SNPs, rs3758853 and rs78356340, which were intronic SNPs, which showed a suggestively significant association with POP in the adjusted model (for both, OR, 3.02; 95% CI, 0.91–9.99; *P* = 0.070) (Table 2).

In the analysis of TIMP family genes, *TIMP2* SNP rs2277698 showed a significant association with POP in the adjusted model (OR, 0.37; 95% CI, 0.16–0.82; *P* = 0.015) and a suggestively significant association with POP in the unadjusted model (OR, 0.50; 95% CI, 0.25–0.98; *P* = 0.062). *TIMP3* SNP rs9862 showed a suggestively significant association with POP in the adjusted model (OR, 1.69; 95% CI, 0.92–3.13; *P* = 0.093). rs2277698 and rs9862 were synonymous SNPs that did not result in amino acid changes. *TIMP4* SNP rs10433537 showed a suggestively significant association with POP in both the unadjusted and adjusted model (OR, 4.83; 95% CI, 1.02–23.13; *P* = 0.058 and OR, 4.16; 95% CI, 0.85–20.31; *P* = 0.078, respectively), which was an intronic SNP (Table 2).

For the analysis of some of the ADAMTS family genes, there was a significant association between *ADAMTS13* SNP rs149586801, which was an intronic SNP, and POP in both the unadjusted and adjusted models (OR, 0.26; 95% CI, 0.07–0.78; *P* = 0.011 and OR, 0.18; 95% CI, 0.05–0.69; *P* = 0.012, respectively). There was also a suggestively significant association between rs1055432 and POP in the adjusted model (OR, 0.48; 95% CI, 0.22–1.09; *P* = 0.080). rs1055432 was a synonymous SNP that did not lead to the amino acid alteration (Table 2). We have also identified a trend toward significance for the *ADAMTS14* SNP rs4747097 and POP (OR, 1.83; 95% CI, 0.93–3.56; *P* = 0.078) in the adjusted model, which was an intronic SNP (Table 2).

**Fig. 1:**
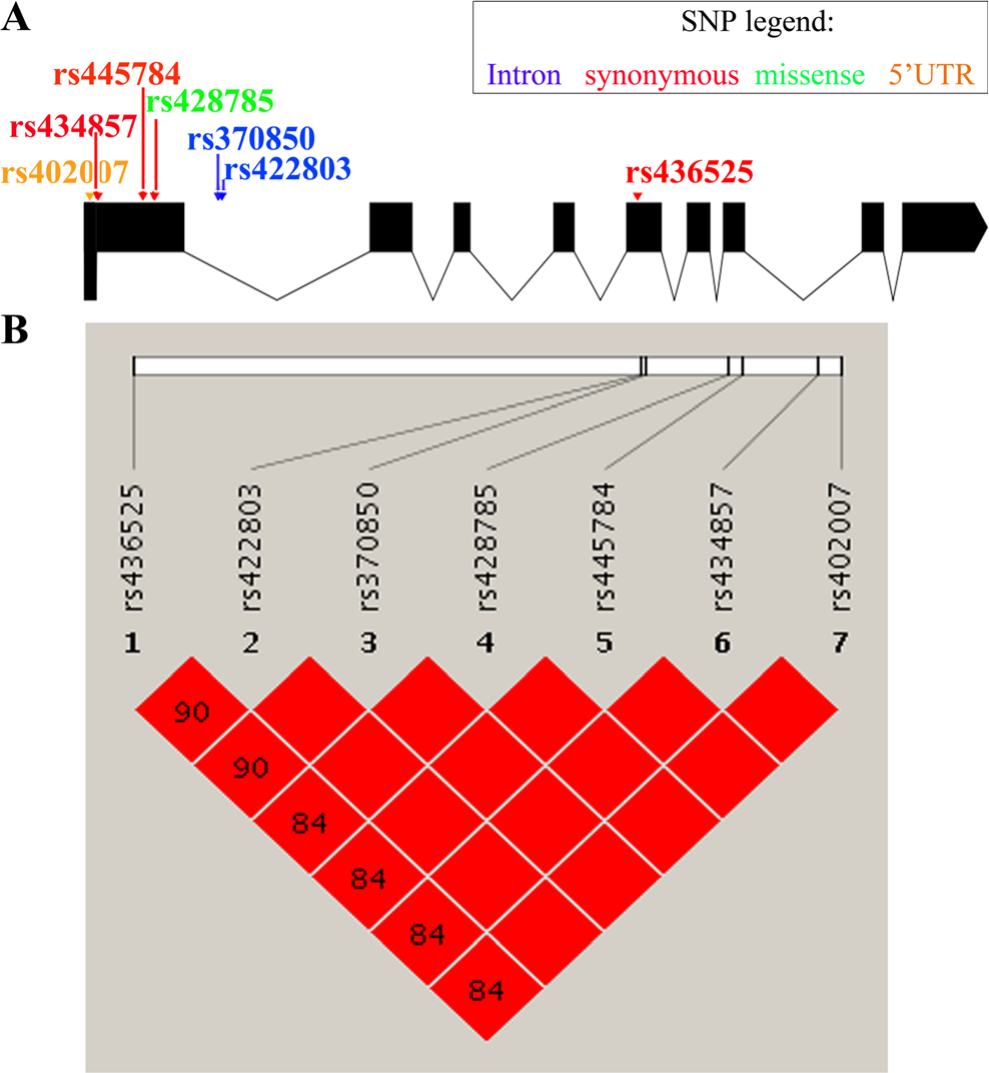
*ADAMTS1* gene structure, indication, and the linkage disequilibrium (LD) of the SNPs. (**A**) Gene structure of *ADAMTS1*. Exons are presented in the vertical black regions. SNPs with a significant or suggestively significant association with POP are indicated by arrows in different colors: synonymous (red), missense (green), introns (blue), and 5’UTR (orange). (**B**) LD graphics for *ADAMTS1* SNPs with r^2^ in each of the boxes. Two SNPs showed greater correlation when the r^2^ value was closer to 1. Boxes with no values meant r^2^ = 1 for perfect LD

In the analysis of *ADAMTS1*, rs370850 and rs422803, which are intronic SNPs, were respectively significantly associated with POP in the adjusted model (for both, OR, 3.71; 95% CI, 1.35–10.15; *P* = 0.011). They also showed a trend toward significance with POP in the unadjusted model (for both, OR, 1.87; 95% CI, 0.94–3.77; *P* = 0.076). rs402007, rs428785, rs434857, and rs445784 were respectively significantly associated with POP in the adjusted model (for all, OR, 2.18; 95% CI, 1.05–4.56; *P* = 0.038). rs402007 was in the 5’UTR region of the *ADAMTS1* gene. rs428785 was a missense SNP. rs434857 and rs445784 were synonymous SNPs that did not lead to amino acid changes. There was also a trend toward significance for the SNP rs436525 and POP in the adjusted model (OR, 1.86; 95% CI, 0.91–3.82; *P* = 0.090). rs436525 was also a synonymous SNPs without amino acid changes (Table 2). These seven SNPs are indicated in the schematic of Fig. 1A where the exons and introns of the *ADAMTS1* gene were described. rs436525 was in good LD with six other SNPs (r^2^ > 0.8), and rs370850, rs422803, rs402007, rs428785, rs434857, and rs445784 were in perfect LD between each other (r^2^ = 1) (Fig. 1B). The missense SNP rs428785 resulted in a substitution of alanine to proline, which might cause the change of the protein secondary structure.

## Discussion

POP is a common female pelvic disorder due to the decline of the pelvic floor supportive tissues. The imbalance of synthesis and degradation of collagen and other ECM components plays a role in the pathogenesis of POP. Such regulation of ECM stability mainly relied on MMPs, which can cleave collagen, elastin, and proteoglycans, etc., and their inhibitors TIMPs [20]. ADAMTSs are enzymes involved in the process of procollagen [16]. Thus, many studies have focused on these ECM remolding genes. In this case-control association study in Chinese women, we sequenced some of the MMP, TIMP, and ADAMTS family genes. To test the association of SNPs and POP, we performed Fisher’s exact tests in the unadjusted model. As we matched case and control groups by age, BMI, and the number of postmenopausal women, and did not match for delivery and pregnancy, which were also crucial risk factors for POP and might be the potential to introduce bias, we additionally conducted logistic regression analysis in the model adjusted for delivery and pregnancy. We tested the results of previous studies on *MMP9* and *10* polymorphisms and further analyzed the association of some other MMP genes, TIMPs, and some ADAMTS genes with risks of POP for the first time. Besides *MMP9* and *10*, our study provided novel evidence that *MMP13*, *TIMP2*, *3*, *4*, and *ADAMTS1, 13*, *14* might be possible candidate genes for POP.

MMPs act on ECM breakdown in multiple physiological and pathological processes, including embryonic development, tissue remodeling, wound repair, angiogenesis, inflammatory processes, and tumor progression [21]. According to the structure of the domain and the type of their substrates, MMPs, with 24 family members in mammals, are classified into several subtypes [22]. Among those, MMP1, MMP8, and MMP13 are interstitial collagenases, which mainly degrade collagens I, II, and III. MMP2 and MMP9 are gelatinases, which can cleave collagens and gelatins [23]. MMP2 can also degrade collagens I, II, and III [24]. MMP3 and MMP10 are stromelysins. In addition to degrading ECM components, MMP3 can activate other MMPs, such as MMP1 [25]. TIMPs are specific inhibitors of MMPs and key regulator for their activities. The proper proportion of MMPs and TIMPs can maintain the dynamic balance of ECM and thus may maintain the stability of the supporting structure of the pelvic floor.

The role of MMPs in the development of POP has been primarily explored in their expression changes as well as their susceptible loci for POP. Previous analysis demonstrated that women with POP showed higher expression levels of MMP1 and MMP8 compared with the control women [26]. The expression of MMP2 and MMP9 were higher in the uterosacral ligament in women with POP [27]. For the analysis of MMP polymorphisms, limited MMPs have been explored. Since the sequencing in this study did not involve the promoter regions of *MMP1*, *3*, and *9* in our target region sequencing approach, we did not verify our data in relation to the previous polymorphisms in the promoter regions of *MMP1*, *3*, and *9*. However, we compared our results to the previous *MMP9* and *10* studies [12–14]. Chen et al. [12] conducted a case-control associated study evaluating three *MMP9* SNPs, rs3918242, rs17576, and rs2250889, and found that rs17576 G allele was a risk site for POP. The authors recruited 92 POP patients at POP-Q stage ≥ II and 152 control women at stage 0–I of Taiwanese women. Wu et al. [13] additionally detected 8 *MMP9* SNPs and found rs3918253 and rs3918256 associated with POP in non-Hispanic white women in 239 cases with stages III and IV and 197 controls with stages 0–I. In our study, we did not find the previously reported *MMP9* SNPs rs3918253 and rs3918256 by Wu et al. [13] in significant association with POP. Chen et al. [12] reported rs17576 was significantly associated with POP; however, Wu et al. [13] and our study both obtained the opposite result. Moreover, for other previously detected *MMP9* SNPs rs3918242, rs2250889, rs3918278, rs2274755, rs17577, rs2236416, and rs3787268 by Wu et al. and Chen et al., we confirmed that these SNPs also showed no association with POP in our study. Besides validating the previous study, we additionally identified a *MMP9* SNP rs3918254 that had a suggestively significant association with POP, which was an intronic SNP. Wang et al. [14] detected a higher MMP10 serum level in the POP group than in the control group and also found *MMP10* SNP rs17435959 genotype G/C was distributed differentially between 91 women with POP at stage ≥ II and 172 control women at stage 0-I of Chinese ancestry. However, we did not find a significant association in the *MMP10* gene in our study. The inconsistency of these studies may partially be attributed to the differences in the recruitment criteria and the characteristics of the women, for example, the race, age, BMI, parity, etc., which were key influencing factors for POP, or in the detection methods.

Studies on other MMP polymorphisms have not been conducted in POP. In this study, besides *MMP9* and *MMP10*, we have sequenced multiple MMP genes and aimed to find the association between these genes and POP. However, we did not observe any positive associations among *MMP1, 2, 3, 8, 10,* and POP, respectively. Additionally, we have found two new *MMP13* SNPs, rs3758853 and rs78356340, which showed borderline significant associations with POP and may have a risk effect for POP.

There were few studies focusing on TIMP family genes and POP. TIMP2 expression level in the POP group was lower than that in the control group [27]. TIMP3 also showed lower levels in the uterosacral ligament in women with POP compared to non-POP women [28]. In our study, we sequenced *TIMP1*, *2*, *3*, and *4* genes for the first time. We found a significant association between a *TIMP2* SNP rs2277698 and POP. Our data suggested that it may show a protective effect for POP development. We also reported a *TIMP3* SNP rs9862 and a *TIMP4* SNP rs10433537 that had a borderline association with POP, which should be further investigated in future research.

The ADAMTS protease family plays crucial roles in ECM remodeling and tissue morphogenesis as well as in inflammation and other physiological and pathological processes [29]. This family consists of 19 members which belong to different subgroups. For example, ADAMTS1 and 8 are the aggrecanases or proteoglycanases [30], ADAMTS2, 3, and 14 are the N-propeptidases of the procollagen [31], and ADAMTS13 is the well-recognized von-Willebrand factor proteinase [32]. ADAMTSs participate in the synthesis of ECM by their effects on the N-propeptidases of the procollagens and in the modification of ECM proteoglycans by their aggrecanase function. However, several studies on ADAMTSs have been performed in relation to the development of POP. One of the ADAMTS members, ADAMTS16, was identified as genome wide suggestive in a genome-wide association study (GWAS) on urgency urinary incontinence (UUI) [33], indicating the involvement of ADAMTS16 in this pelvic floor dysfunction. In our study, we found a significant association among *ADAMTS1* SNPs rs370850, rs422803, rs402007, rs428785, rs434857, and rs445784 and POP, which may be risk factors related to POP, and a significant association between *ADAMTS13* SNP rs149586801 and POP, which showed potential protective effects for this disease. We also found three additional SNPs, *ADAMTS1* SNP rs436525, *ADAMTS13* SNP rs1055432, and *ADAMTS14* SNP rs4747097, which have suggestively significant associations with POP. Additionally, *ADAMTS1* SNP rs428785 is a missense SNP and can lead to a substitution of alanine to proline, which might exhibit a benign and tolerant effect predicted by PolyPhen-2 and SIFT. Therefore, *ADAMTS1*, *ADAMTS13*, and *ADAMTS14* could be novel candidate genes for POP, and this needs further validation.

Our study had several strengths. As previous studies were few and focused on certain limited SNPs of a gene, we sequenced several MMP, TIMP, and ADAMTS family genes by target region sequencing technique to assess the entire genes with their coding regions. Second, we not only validated the data of the previously identified SNPs in *MMP9* and *10*, but also analyzed the association between *MMP1, 2, 3, 8, and 13* and POP for the first time. Additionally, the polymorphisms of *TIMP* and some *ADAMTS* family genes have been first studied in POP, suggesting new susceptible SNPs. Third, we focused on unrelated Chinese women from mainland China in this candidate gene association study, choosing extreme symptoms of severe POP with POP-Q stages III and IV, tying to improve the detection of the potential variants.

However, the main limitation of our study was the small sample size. Since our nationwide epidemiological survey in mainland China found that the prevalence of POP at stages III and IV was 2.04% [34], this made it quite difficult to select women under this stage limitation within a certain period of time. Thus, power may be limited in this study because of the relatively small sample size. Although we accepted all variants with a minor allele frequency of 5%, variants identified as being significant or suggestive had a much higher allele frequency than 5%. If we only consider the variants of significant associations (allele frequencies ranging from 0.07–0.47, OR ranging from 0.18–3.71), the power can make our data use a reference to some extent according to the different OR value of these SNPs. Hypotheses regarding the pathophysiology of POP include abnormal synthesis or degradation of ECM, and we provided new evidence on *MMP*, *TIMP*, and *ADAMTS* polymorphisms for future investigation of the involvement of these candidate genes in the etiology of POP. The genetic contributions to POP remain poorly understood. Additional work needs to be done to provide further validation of POP predisposition variants in a variety of different populations to establish the role of these genes in the pathogenesis of prolapse [5].

## Conclusion

MMP, TIMP and ADAMTS family genes are crucial for ECM synthesis, modification, and metabolism. In this study, we provide initial evidence that the genetic variants in these genes may have an association with POP. We identified several susceptible SNPs in the *MMP9* and *13*, *TIMP2*, *3*, and *4* and *ADAMTS1*, *13*, and 14 genes in Chinese groups, preliminarily revealing the underlying mechanisms for the pathophysiology of this common disease.

## Supplementary Information

Table S1 All of the variants with a minor allele frequency of 5% (XLS 66 kb)

## Abbreviations

POP: Pelvic organ prolapse
COL1A1: Collagen type I alpha
COL3A1: Collagen type III alpha 1
LAMC1: Laminin gamma-1
MMP: Matrix metalloproteinase
ECM: Extracellular matrix
TIMP: Tissue inhibitors of metalloproteinase
ADAMTS: A disintegrin and metalloproteinase with thrombospondin motif
SNP: Single-nucleotide polymorphism
POP-Q: Pelvic organ prolapse quantification
BWA: Burrows-Wheeler Aligner
MAF: Minor allele frequency
OR: Odds ratio
CI: Confidence interval
LD: Linkage disequilibrium
BMI: Body mass index
GWAS: Genome-wide association study
UUI: Urgency urinary incontinence
